# Determination of the optimal time of vaccination against infectious bursal disease virus (Gumboro) in Algeria

**DOI:** 10.4102/ojvr.v82i1.887

**Published:** 2015-04-30

**Authors:** Omar Besseboua, Abdelhanine Ayad, Hama Benbarek

**Affiliations:** 1 Department of Agricultural Sciences, University M. Istambouli, Algeria; 2 Department of Environment and Biological Sciences, University A. Mira, Algeria; 3 Laboratory of Research on Local Animal Products, Veterinary Institute, Ibn Khaldoun University, Algeria

## Abstract

This study was conducted to determine the effect of maternally derived antibody (MDA) on live vaccine against infectious bursal disease. A total of 140 chicks selected from vaccinated parent stock were used in this investigation. In a preset vaccination schedule, blood samples were collected to check for the actual effect. It was noticed that on day 1 the chicks contained a high level (6400.54 ± 2993.67) of maternally derived antibody that gradually decreased below a positive level within 21 days (365.86 ± 634.46). It was found that a high level of MDA interferes with the vaccine virus, resulting in no immune response. For better immune response, it is suggested that the chickens should be vaccinated at day 21, as the uniformity of MDA is poor (coefficient of the variation [CV] > 30%), and boosted at day 28. Indeed, two vaccinations are necessary to achieve good protection against infectious bursal disease virus of the entire flock.

## Introduction

Infectious bursal disease (IBD), also known as Gumboro disease, which is caused by a member of the *Birnaviridae* family, genus *Avibirnavirus* (Murphy *et al.* 1999), is responsible for an acute and highly contagious viral infection for young chicks (Lukert & Saif 1991). Devastating outbreaks of the disease have been reported in many parts of the world (Farooq *et al.* 2003) and recently in the United States of America (Jackwood *et al.* 2009). The importance of the disease is reflected by the high mortality (Anjum, Sabri & Jamshidi 1994), reduced productivity amongst infected chicks (Shane, Lasher & Paxton 1994) and increased susceptibility to other infections. Accordingly, chickens also develop a poor immune response to vaccination against other pathogens (Ali, Abdalla & Mohammed 2004; Sharma *et al.* 2000). Hermann, Rafiqul Islam and Raue (2003) indicated that the mortality rate may range from 1% to 50% in the classical form of the outbreak. The same authors reported that infection may result in up to 50% morbidity, but mortality is rarely above a threshold of 3% in flocks that are 3–6 weeks old. The disease causes significant economic losses in the poultry industry worldwide (Mahmood *et al.* 2006; Müller *et al.* 2012; Uddin *et al.* 2010).

The control of IBD was found to depend on appropriate immunisation schedules and maintenance of good hygienic conditions on the farm (Farooq *et al.* 2003). Vaccinating breeding hens with live attenuated or inactivated virus vaccine could control the disease more effectively. Induced antibodies are transferred to the young chicks via the egg yolk. They protect the newly hatched chicks for the critical first few weeks of life (Wyeth & Cullen 1976). In spite of the extensive use of vaccines, farmers still have to contend with Gumboro disease. To meet the requirements of our farmers, several live and killed vaccines used against IBD are frequently imported to Algeria from abroad. Many manufacturing companies have their own vaccine specifications. They are used on commercial poultry farms at days 7 and 14 regardless of the status of maternally derived antibody (MDA) in offspring and its consequent effect on vaccination with live vaccine.

The present study was carried out with a specific focus on three objectives: (1) detecting the persistence of MDA in progeny from vaccinated parent stock, (2) determining the effect of vaccination with live vaccine against Gumboro disease in day-old chicks and (3) proposing the optimal vaccination schedule against IBD virus (IBDV).

## Materials and methods

### Chicks and sampling

This study was conducted from February to March 2008 at Misserghin farm in Oran, western Algeria. The experimental protocol was approved by the Faculty Council of the University M. Istambouli, Mascara, Algeria. Blood sampling of the chicks was carried out according to the rules of good veterinary practice under farm conditions. A total of 140 day-old chicks of classic Institut de Sélection Animale (ISA) derived from vaccinated parent stock were used in this study. The chicks were reared for 6 weeks in a well-ventilated poultry house, maintaining all the hygienic measures applicable.

The non-vaccinated chicks were divided into six groups (A, B, C, D, E and F) of 20 individuals each. The chicks immunised on day 5 were allocated to Group G (*n* = 20). Blood samples were collected from the non-vaccinated groups after slaughter at days 1 and 7 and from the brachial vein at days 10, 14, 21 and 28. In Group G post-vaccinal plasma was collected from the brachial vein 12 days after vaccination.

Blood samples were collected into tubes containing heparin. The plasma obtained by centrifugation (1500 x g for 15 min) was stored at -20 °C prior to testing. Enzyme-linked immunosorbent assay (ELISA) was used for the detection of either MDA or antibody induced by vaccination. The MDA-or IBDV-specific antibody titres in vaccinated and non-vaccinated chicks were determined in the plasma by ELISA (CIVTEST AVI IBD, Hipra, Girona) performed in duplicate as described previously (Alam *et al.* 2002). The test sample used consisted of a mixture of 5 μL of plasma with 2.5 mL diluents (i.e. 1:500) contained in the ELISA kit.

### Calculation of results

The presence or absence of anti-IBDV antibody was determined by comparing the absorbance (450) value of the unknown to that of the positive control. The standardised positive control represented a significant IBDV antibody level in chicken plasma (absorbance > 0.6). For analysis of the data, an S/P ratio is required (sample value related to positive control value). The following formula was applied using mean absorbance values for both controls and paired samples and the antibody titre was calculated using the equation provided in the ELISA kit:

Negative control (NC) mean:

$$N\bar{CX}=\frac{(wellA\text{1(450) + }wellA\text{2(450))}}{\text{2}}$$ [Eqn 1]

Positive control (PN) mean:

$$P\bar{CX}=\frac{(wellA\text{3(450) + }wellA\text{4(450))}}{\text{2}}$$ [Eqn 2]

S/P ratio:

$$S/P=\frac{SAMPLEABSORBANCE-NCABSORBANCE}{PCABSORBANCE-NCABSORBANCE}$$ [Eqn 3]

Titre relates S/P at a 1:500 dilution to an end point titre:

$$\log_{10}\text{titre = 1}{\text{.35(log}}_{10}\text{ S/P)+3}.\text{52}$$ [Eqn 4]

$${\text{titre = antilog of log}}_{10}\text{ titre}$$ [Eqn 5]

Plasma samples with S/P ratios ≤ 0.2 were considered negative, whereas S/P ratios > 0.2 (titres > 455) were assumed positive, indicating either vaccination or exposure to IBDV.

### Statistical analysis

Calculations were performed using Statistica software (Statsoft, version 6). The data were expressed as mean ± s.d. (%). The coefficient of the variation (CV%) of the MDA level at different ages after hatching was calculated.

## Results

The results of ELISA tests for MDA performed on the blood of chicks obtained from vaccinated parent stock (Table 1) revealed that the antibodies of non-vaccinated chicks decreased continuously from day 1 to day 28 after hatching. The level of MDA was high at days 1 and 7 (6400.54 ± 2993.67 and 6294.18 ± 2525.21, respectively). The MDA values decreased slowly at days 10 and 14 but sharply at days 21 and 28 (365.86 ± 634.46 and 188.50 ± 214.89, respectively). Note that there is a lack of homogeneity in the data. The correlation coefficient (R^2^) between the MDA and various ages of chicken from vaccinated parent stock was 0.92.

**TABLE 1:** Maternally derived antibody titres determined by enzyme-linked immunosorbent assay at different ages of non-vaccinated chicks from vaccinated parent stock (days 1, 7, 10, 14, 21 and 28).

Days	OD sample (mean ± s.d.)	S/P ratio (mean ± s.d.)	Amount of MDA (mean ± s.d.)
NC	0.18	-	-
PC	0.6	-	-
1 (Group A)	0.86 ± 0.24	1.63 ± 0.58	6400.54 ± 2993.67
7 (Group B)	0.85 ± 0.21	1.61 ± 0.51	6294.18 ± 2525.21
10 (Group C)	0.74 ± 0.27	1.33 ± 0.63	4890.05 ± 3102.29
14 (Group D)	0.64 ± 0.31	1.09 ± 0.73	3709.02 ± 3196.36
21 (Group E)	0.26 ± 0.10	0.19 ± 0.23	365.86 ± 634.46
28 (Group F)	0.23 ± 0.05	0.12 ± 0.08	188.50 ± 214.89

OD, optical density; MDA, maternally derived antibody; NC, negative control; PC, positive control; S/P, sample absorbance-NC absorbance/PC absorbance-NC absorbance; s.d., standard deviation.

*n* = 20 birds examined in each group.

Results showed that the amount of antibodies in the chicks from immune hens subjected to vaccination on day 5 was reduced by one-fifth after 12 days compared with the amount of MDA detected in the chicks at day 1 (Table 2). However, the amount of antibodies still remained above the positive level (1242.34 ± 1139.69). As indicated above, according to the ELISA antibody test kit, an S/P ratio ≤ 0.2 should be considered negative whereas an S/P ratio > 0.2 is positive for antibody.

**TABLE 2:** Immune response in chicks from vaccinated parent stock vaccinated (day 5) with live vaccine (Group G).

Days (Group G)	PC	NC	Day before vaccination (day 1) (mean ± s.d.)	Day after vaccination (day 17) (mean ± s.d.)
Sample OD	0.6	0.18	0.86 ± 0.24	0.38 ± 0.13
S/P ratio	-	-	1.63 ± 0.58	0.48 ± 0.32
Amount of MDA	-	-	6400.54 ± 2993.67	1242.35 ± 1139.69

PC, positive control; NC, negative control; OD, optical density; MDA, maternally derived antibody; S/P, sample absorbance-NC absorbance/PC absorbance-NC absorbance; s.d., standard deviation.

*n* = 20 birds examined in Group G.

CV, coefficient of the variation; MDA, maternally derived antibody.

**FIGURE 1:**
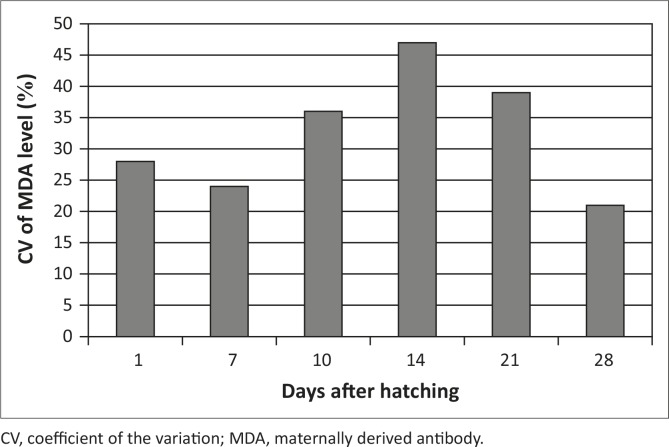
Coefficient of variation (CV%) of the maternally derived antibody titres of non-vaccinated chicks (Groups A, B, C, D, E and F) from vaccinated parent stock at different ages (days 1, 7, 10, 14, 21 and 28).

CV, coefficient of the variation; MDA, maternally derived antibody.

The uniformity of day-old broiler chicks can be estimated by serological profiling and expressed as a percentage coefficient of variation (CV%). Good and poor uniformity are coefficients of variation < 30 or > 30%, respectively. Based on that reference value, the flocks had the best uniformity of the MDA with a CV of 28% and 24% at days 1 and 7, respectively (Figure 1). After that the CV increased gradually, which meant the MDA levels at days 10, 14, and 21 attained values of 36%, 47% and 39%, respectively. Finally, it decreased at day 28 to reach a value of 21%.

## Discussion

The level of MDA is high for chicks derived from immune hens at an early age, but it decreases rapidly after day 21. It is noteworthy that antibodies were present in the blood of the chicks until the end of the experiment, that is, day 28. Similar observations were reported by Zaheer, Naeem and Malik (2003). Wisniewska and Stosik (1999) demonstrated traces of MDA in the blood of chicks until days 11–19 and later at day 23 after hatching. Other researchers claimed that the antibodies persist up to day 28 (Hitchner 1971), day 29 (Wyeth & Cullen 1979), day 30 (Iordanides, Koumpate & Artopois 1991) and day 20 after hatching (Al Mayah & Al Mayah 2013; Chansiripornchai & Sasipreeyajan 2009). These substantial differences could be ascribed to the amount of antibodies transferred from hen to chick through the egg (Hamal *et al.* 2006; Rai *et al.* 2005). Rao *et al.* (1987) concluded that the MDA depends on the quantity of egg yolk.

Lukert and Saif (1997) noticed that the half-life of MDA to IBDV is between 3 and 5 days. Similarly, other studies have reported that the half-life MDA to IBD in chicks was 3.46 days (Saijo & Higashihara 1998) and decreased every 4 days (Gardin 1994). Others reported that the rate of decline was by about half every 5 days (Alam *et al.* 2002; Shrestha *et al.* 2003) and between 4 and 5 days (Sheku 2013). In newly hatched layer-type chicks, MDA exhibits a linear or curvilinear decline with a mean half-life of 5 to 6 days (Müller *et al.* 2012). Fahey, Crooks and Fraser (1987) reported a half-life of 6.7 days for IBDV-specific MDA. It is generally thought that the half-life of MDA in broiler lines is much shorter, approximately 3 days (Block *et al.* 2007). Data from this study revealed that the decrease of MDA to IBDV is variable during the growing period. This divergence may be explained by the influence on the half-life of MDA of the vaccine type, its time of application in hens (Alam *et al.* 2002) and probably the immune status of the hen (Kouwenhoven & Van den Bos 1992). Moreover, whilst the antibody titres may not vary greatly amongst hens in a single flock of similar age, the offspring of different vaccinated flocks may show different IBDV MDA titres. When offspring of different parent flocks are raised together, this may result in different levels of MDA and compartmentalisation of the herd into individuals with low or high susceptibility to virulent IBDV (Tsukamoto *et al.* 1995). Under field conditions, however, the decay pattern of IBDV-specific MDA proved to be more complex, as it depends largely on initial antibody levels, which may vary between batches and also within a batch, making it difficult to predict the optimal time for vaccination (De Wit 1998).

The results of this study correspond with those reported by Zaheer and Saeed (2003), who noted that the susceptible time is when the level of antibodies is lower than the threshold of the protection level (days 21–35). Researchers confirmed that the level of MDA of chicks (56.6%) was below the level of protection after 3 weeks (Cardinale *et al.* 1998) or 15–20 days (Alam *et al.* 2002) after hatching. All breeds of chickens from vaccinated parent stock were found to contain high levels of MDA at day 1, which decreased gradually below the protection level within 15–20 days after hatching (Shrestha *et al.* 2003). Well before this, Jordon and Pattison (1996) demonstrated that it decreased below the level of protection within a period of 2.5–3.5 weeks after hatching. In contrast, Phatak (2002) noted that the amount of antibodies often decreased within 7–14 days from hatching. The discrepancy most probably reflects the use of different types of vaccine and vaccination schedules (Alam *et al.* 2002). Al Mayah and Al Mayah (2013) concluded that intermediate plus vaccines induced higher antibody titres than other vaccines, although some intermediate vaccines induced similar titres. The results of the present study were also in agreement with those reported by Amer *et al.* (2007), who stated that the ELISA antibody titres from vaccination with intermediate vaccines were the lowest at all intervals whilst the titres of intermediate plus vaccine were the highest. Therefore the time at which the level of antibodies is below the level of protection for chicks is variable.

Early vaccine failed to stimulate the immune system in the chicks because maternal antibody reacts with live vaccine virus and becomes neutralised or interferes with MDA (Zhuo *et al.* 1998). Several studies under laboratory conditions have indicated that high MDA at the time of IBDV vaccination might interfere with the vaccine response, neutralises the vaccine virus and delays or even prevents the induction of humoral immunity (Hair-Bejo *et al.* 2004; Jung 2006; Morães *et al.* 2005). This means that the vaccination in the first days failed to offer the chick any protection against disease. Nevertheless, an increase in titre was observed when vaccination was performed at 14 days (Knezevic *et al.* 1999), as also observed by Kumar, Singh and Prasad (2000) using a quantitative agar gel precipitation test.

As shown in Table 2, the MDA to IBDV was high before vaccination but decreased after vaccination. However, the amount of antibodies remained above the protection level (1242.34 ± 1139.69). Similar results were reported by Alam *et al.* (2002); the chicks did not achieve immunity against disease at day 7 after hatching. This indicated that MDA neutralised the vaccine virus injected at day 5. The level of MDA decreases over time, especially at 2–3 weeks of age (Rautenschlein, Yeh & Sharma 2002), which reduces neutralisation or interference with live vaccine virus that achieves good immunisation of flock.

Sahar, Ali Mahasan and Rahman (2004) recommended vaccinating chickens at an age of 2 weeks with intermediate strains of IBD and boosting them with the ‘hot’ vaccine at an age of 3 weeks in a closed system. Suzuki *et al.* (2009) reported estimated optimal vaccination timings against IBDV of each flock at the three sampling time points between 16 and 24 days of age. Similarly, Block *et al.* (2007) indicated that the optimal vaccination time was between 17 and 23 days post-hatch based on the Deventer formula, whilst Lone *et al.* (2012) suggested that broiler chicks vaccinated at days 8, 15 and 23 with live attenuated vaccine or live attenuated vaccine followed by inactivated vaccine at days 8 and 21 could be adequately protected against the virulent form of IBDV. Furthermore, Al-Mufarrej (2013) observed that chickens vaccinated at days 10 or 18 showed better immune response to IBDV vaccination.

In this study, the MDA amount was approximately equal to the amount of antibodies (3709.02 ± 3196.36) for chicks at day 14. This suggested that vaccination at day 14 is not appropriate because the amount of maternal antibodies is above the level of protection (Table 1). These results agree with the data obtained by Cardinale *et al.* (1998), where the level of antibodies for chicks was above the level of protection at day 14, although Hair-Bejo *et al.* (2004) reported that vaccination was successful at day 14. A lack of antibodies that should ensure protection for chicks used could be the cause. On the other hand, non-immune chicks become vulnerable to Gumboro disease after 3 weeks, according to reports by Alam *et al.* (2002) that show that vaccinating chicks from immune hens did not achieve immunisation against the disease at day 7 after hatching.

In the present investigation, the amount of antibodies was high in chicks on the first day, which could be ascribed to a failure of vaccination. The level of antibodies remained high in chicks up until day 15. Van den Berg and Meulemans (1991) noticed that chicks vaccinated on day 14 did not achieve immunity as opposed to those vaccinated at days 21–28, and recommended vaccination at day 21 because at that time the level of MDA is below the level of protection. Similarly, Sarachai, Chansiripornchai and Sasipreeyajan (2010) reported that the appropriate age for intermediate plus vaccination is 22 days. However, Moiforay (2013) observed that the appropriate time to implement active vaccination without risk of vaccine failure or incidence of infection was between days 24 and 26 post hatching.

It was observed in this study that chicks on day 1, 7 and 14 contained high level of MDA that declined gradually to below a positive level within 21 days. As shown in Table 1, the flock had the worst uniformity of MDA at day 21, which may support the recommendation to vaccinate twice with a two-day interval for the protection of the entire flock. Suzuki *et al.* (2009) suggested that the flock had the best uniformity of MDA only when vaccinated once at day 22.

The results of this study showed the best uniformity of MDA at day 28 for the entire flock, although it is slightly below the protection level. Therefore, it was deduced that vaccination was successful and achieved good immunisation for chicks. These results agree with those reported by Van den Berg and Meulemans (1991). A high variation in MDA levels between birds in a flock can make it advisable to vaccinate a broiler flock twice to induce homogeneous protection (McIlroy *et al.* 1992). Thus, uniformity of the MDA titre distribution is related to the number of vaccinations required (Suzuki *et al.* 2009). Vaccination programmes play an important role in providing adequate protection (Chansiripornchai & Sasipreeyajan 2009; De Wit & Baxendale 2013) but may vary from country to country and area to area (Block *et al.* 2007).

## Conclusion

The present study clearly shows that a high level of maternally derived antibody interferes with the vaccine, resulting in no immune response. However, re-vaccination induces an immune response, particularly when carried out at days 21 and 28. Indeed, two vaccinations could be recommended to achieve good protection against infection by bursal disease virus in a flock.

## References

[CIT0001] Alam J., Rahman M.M., Sil B.K., Khan M.S.R. & Sarker M.S.K., 2002, ‘Effect of maternally derived antibody on vaccination against infectious bursal disease (Gumboro) with live vaccine in broiler’, *International Journal of Poultry Science* 1(4), 98–101. http://dx.doi.org/10.3923/ijps.2002.98.101

[CIT0002] Ali A.S., Abdalla M.O. & Mohammed M.E.H., 2004, ‘Interaction between Newcastle disease and infectious bursal disease vaccines commonly used in Sudan’, *International Journal of Poultry Science* 3, 300–304. http://dx.doi.org/10.3923/ijps.2004.300.304

[CIT0003] Al Mayah I.M.D. & Al Mayah A.A.S., 2013, ‘Antibody response of broiler chickens against eight commercial infectious bursal disease live vaccines tested by ELISA’, *Mirror of Research in Veterinary Sciences and Animals* 2(2), 1–7.

[CIT0004] Al-Mufarrej S.I.A., 2013, ‘A comparative study of two vaccines against infectious bursal disease in newly hatched broiler chickens’, *Agricultural Journal* 8, 101–105.

[CIT0005] Amer M.M., El-Bayomi K.M., Kotkat M., Abd A., Wafaa A., Shakal M.A. et al., 2007, ‘The efficacy of live infectious bursal disease vaccines in commercial 10 days old chicks’, *Proceedings of the 5th Scientific Conference*, Faculty of Veterinary Medicine, Beni Suef University, Egypt, Nov-09, 2007, pp. 23–33.

[CIT0006] Anjum A.D., Sabri G.S. & Jamshidi K., 1994, ‘Occurrence spread and control of infectious bursal disease in Pakistan’, *Proceedings of 1st PPAPVMA Punjab, International Poultry Conference*, Punjab, India, Mar-Apr, pp. 57–59.

[CIT0007] Block H., Meyer-Block K., Rebeski D.E., Scharr H., De Wit S., Rohn K. et al., 2007, ‘A field study on the significance of vaccination against infectious bursal disease virus (IBDV) at the optimal time point in broiler flocks with maternally derived IBDV antibodies’, *Avian Pathology* 36(5), 401–409. http://dx.doi.org/10.1080/03079450701589175 17899465 10.1080/03079450701589175

[CIT0008] Cardinale E., Arbelot B., Kaboret Y., Dayon J.F., Biaou C. & Bada Algom O., 1998, ‘La maladie de Gumboro dans les élevages semi-industriels de la région de Dakar’ [Gumboro disease in semi-intensive poultry farms of Dakar area], *Revue d*’É*levage et de Médecine Vétérianaire des Pays Tropicaux* 51(4), 293–296.

[CIT0009] Chansiripornchai N. & Sasipreeyajan J., 2009, ‘Comparison of the efficacy of the immune complex and conventionally live vaccine in broilers against infectious bursal disease infection’, *Thailand Journal of Veterinary Medicine* 39(2), 115–120.

[CIT0010] De Wit J.J., 1998, ‘Gumboro disease: Estimation of optimal time of vaccination by the Deventer formula’, *Polish Veterinary Journal* 3, 19–22.

[CIT0011] De Wit J.J. & Baxendale W., 2013, *Gumboro*, viewed 03 March 2013, from http://www.gumboro.com/control/vaccination/index.asp

[CIT0012] Fahey K.J., Crooks J.K. & Fraser R.A., 1987, ‘Assessment by ELISA of passively acquired protection against infectious bursal disease virus in chickens’, *Australian Veterinary Journal* 64, 203–207. http://dx.doi.org/10.1111/j.1751-0813.1987.tb15182.x 2823764 10.1111/j.1751-0813.1987.tb15182.x

[CIT0013] Farooq M., Durrani F.R., Imran N., Durrani Z. & Chand N., 2003, ‘Prevalence and economic losses due to infectious bursal disease in broilers in Mirpur and Kolti districts of Kashmir’, *International Journal of Poultry Science* 2(4), 267–270. http://dx.doi.org/10.3923/ijps.2003.267.270

[CIT0014] Gardin Y., 1994, ‘Application of an invasive vaccine under controlled condition to solve Gumboro disease problems in France’, *Proceedings of the international symposium on infectious bursal disease and chicken infectious anaemia*, Rauischholzhauzen, Germany, Jun-24, 1994, pp. 286–304.

[CIT0015] Hair-Bejo M., Ng M.K. & Ng H.Y., 2004, ‘Day old vaccination against infectious bursal disease in broiler chickens’, *International Journal of Poultry Science* 3(2), 124–128. http://dx.doi.org/10.3923/ijps.2004.124.128

[CIT0016] Hamal K.R., Burgess S.C., Pevzner I.Y. & Erf G.F., 2006, ‘Maternal antibody transfer from dams to their egg yolks, egg whites and chicks in meat lines of chickens’, *Poultry Science* 85, 1364–1372. http://dx.doi.org/10.1093/ps/85.8.1364 10.1093/ps/85.8.1364 16903465

[CIT0017] Hermann M., Rafiqul Islam M.D. & Raue R., 2003, ‘Research on infectious bursal disease: The past, the present and the future’, *Veterinary Microbiology* 97, 153–165. http://dx.doi.org/10.1016/j.vetmic.2003.08.005 14637046 10.1016/j.vetmic.2003.08.005

[CIT0018] Hitchner S.B., 1971, ‘Persistence of parental infectious bursal disease antibody and its effects on susceptibility of young chickens’, *Avian Diseases* 15, 894–900. http://dx.doi.org/10.2307/1588880 5159555

[CIT0019] Iordanides P., Koumpate M. & Artopois E., 1991, ‘Role of maternal antibodies in preventing IBD in chicks in the first week of life’, *Delteonten kitenaiatrikes Elareias* 42, 245–249.

[CIT0020] Jackwood D.J., Sommer-Wagner S.E., Stoute A.S., Woolcock P.R., Crossley B.M., Hietala S.K. et al., 2009, ‘Characteristics of a very virulent infectious bursal disease from California’, *Avian Diseases* 53(4), 592–600. http://dx.doi.org/10.1637/8957-061109-Reg.1 20095162 10.1637/8957-061109-Reg.1

[CIT0021] Jordon F.T.W. & Pattison M., 1996, ‘Infectious bursal disease (Gumboro disease)’, in Jordan F.T.W. & Pattison M. (eds.), 1996, *Poultry diseases*, 4th edn., pp. 199–203, Philadelphia, Saunders.

[CIT0022] Jung A., 2006, ‘Pathogenesestudie eines intermediärvirulenten Gumborovirus in spezifiziert-pathogen-freien (SPF) Hühnern und kommerziellen Broiler’, PhD Thesis, University of Veterinary Medicine, Hanover, Germany.

[CIT0023] Knezevic N., Sekler M., Veljovic L.J., Kozlina B. & Rodic J., 1999, ‘First experiences with poulvac R Bursine-2 vaccine against Gumboro disease’, *Proceedings of the 8th Yugoslav Symposium on Poultry Production, Sokobanja*, Yugoslavia, Oct-09, 1999, pp. 8–9.

[CIT0024] Kouwenhoven B. & Van den Bos J., 1992, ‘Control of very virulent IBD in the Netherlands with the so-called hot vaccines’, *Proceedings of the 19th World Poultry Congress*, Amsterdam, Netherlands, Sep-24, 1992, pp. 465–468.

[CIT0025] Kumar K., Singh K.C.P. & Prasad C.B., 2000, ‘Immune response to intermediate strain IBD vaccine at different levels of maternal antibody in broiler chickens’, *Tropical Animal Health and Production* 32, 357–360. http://dx.doi.org/10.1023/A:1005225501513 11147275 10.1023/a:1005225501513

[CIT0026] Lone N.A., Rehmani S.F., Khan T.A. & Kazmi S.U., 2012, ‘Efficacy of live attenuated and inactivated oil emulsion infectious bursal disease virus vaccines in broiler chicks’, *Pakistan Veterinary Journal* 32(4), 539–542.

[CIT0027] Lukert P.D. & Saif Y.M., 1991, ‘Infectious bursal disease’, in Calnek B.W., Barnes H.J., Beard C.W., Reid W.M. & Yoder, Jnr. H.W. (eds.), *Diseases of poultry*, 9th edn, pp. 648–663, Iowa State University Press, Ames.

[CIT0028] Lukert P.D. & Saif Y.M., 1997, ‘Infectious bursal disease’, in Calnek B.W., Barnes H.J., Beard C.W., McDougald L.R. & Saif Y.M. (eds.), *Diseases of poultry*, 10th edn, pp. 721–738, Iowa State University Press, Ames.

[CIT0029] Mahmood M.S., Siddique M., Hussain I., Khan A. & Mansoor M.K., 2006, ‘Protection capacity of recombinant plasmid DNA vaccine containing VP2 gene of very virulent infectious bursal disease virus in chickens’, *Vaccine* 24, 4838–4846. http://dx.doi.org/10.1016/j.vaccine.2006.03.016 16600440 10.1016/j.vaccine.2006.03.016

[CIT0030] McIlroy S.G., Goodall E.A., Bruce D.W., McCracken R.M. & McNulty M.S., 1992, ‘The cost benefit of vaccinating broiler flocks against subclinical infectious bursal disease’, *Avian Pathology* 21, 65–76. http://dx.doi.org/10.1080/03079459208418819 18670916 10.1080/03079459208418819

[CIT0031] Moiforay S.K., 2013, ‘Appropriate time of vaccination against infectious bursal disease virus in layer chicks by Elisa in single dilution’, *Research Journal of Agricultural and Environmental Management* 2(4), 111–116.

[CIT0032] Morães H.L.S., Salle C.T.P., Nascimento V.P., Salle F.O., Rocha A.C.G.T., Souza G.F. et al., 2005, ‘Infectious bursal disease: Evaluation of maternal immunity and protection by vaccination of one-day old chicks against challenge with very virulent virus isolate’, *Brazilian Journal of Poultry Science* 7, 51–57.

[CIT0033] Müller H., Mundt E., Eterradossi N. & Islam M.R., 2012, ‘Current status of vaccines against infectious bursal disease’, *Avian Pathology* 41(2), 133–139. http://dx.doi.org/10.1080/03079457.2012.661403 22515532 10.1080/03079457.2012.661403

[CIT0034] Murphy F.A., Gibbs E.P., Horinek M.C. & Studdert M.J., 1999, ‘Birnaviridae’, in Murphy F.A., Gibbs E.P.J., Horzinek M.C. & Studdert M.J. (eds.), *Veterinary Virology*, 3rd edn, pp. 405–409, Academic Press, London.

[CIT0035] Phatak R.K., 2002, ‘Vaccination failures and their solutions’, *Italian Journal of Animal Science* 2, 157–162.

[CIT0036] Rai M.F., Khan S.A., Aslam A. & Saeed K., 2005, ‘Effects of yolk sac infection in chicken’, *Avian Poultry Biology Reviews* 16(2), 87–93. http://dx.doi.org/10.3184/147020605783438804

[CIT0037] Rao A.S., Chetty M.S., Prasad V.L.K., Reddy P.B., Reddy G.V.B. & Reddy B.D., 1987, ‘Persistence of maternal antibodies against Newcastle disease virus in chicks from immune parents and its effect on vaccination’, *Indian Journal of Comparative Microbiology and Immunology Infectious Diseases* 8, 105–110.

[CIT0038] Rautenschlein S., Yeh H.Y. & Sharma J.M., 2002, ‘The role of T cells in protection by an inactivated infectious bursal disease virus vaccine’, *Veterinary Immunology and Immunopathology* 89, 159–167. http://dx.doi.org/10.1016/S0165-2427(02)00202-7 12383647 10.1016/s0165-2427(02)00202-7

[CIT0039] Sahar M.O., Mahasin Ali, Rahman A.S., E.A., 2004, ‘Residual pathogenic effects of infectious bursal disease vaccines containing intermediate and hot strains of the virus in broiler chickens’, *International Journal of Poultry Science* 3(6), 415–418. http://dx.doi.org/10.3923/ijps.2004.415.418

[CIT0040] Saijo K. & Higashihara M., 1998, ‘Optimal time of initial administration of live vaccine for IBD in chicks with maternally derived antibody’, *Journal of the Japanese Veterinary Medical Association* 51, 647–651. http://dx.doi.org/10.12935/jvma1951.51.647

[CIT0041] Sarachai C., Chansiripornchai N. & Sasipreeyajan J., 2010, ‘Efficacy of infectious bursal disease vaccine in broiler chickens receiving different vaccination programs’, *Thailand Journal of Veterinary Medicine* 40(1), 9–14.

[CIT0042] Shane S.M., Lasher H.N. & Paxton K.W., 1994, ‘Economic impact of infectious bursal disease and prevalence of antigenic variation for protection in infectious bursal disease’, *Proceedings of the 2nd International Symposium on infectious bursal to disease (IBD) and chicken infectious anemia (CIA)*, Rauischholzhausen, Germany, Jun-24, 1994, pp. 196–203.

[CIT0043] Sharma J.M., Kim I.J., Rautenschlein S. & Yeh H.Y., 2000, ‘Infectious bursal disease virus of chicken: Pathogenesis and immunosuppression’, *Developmental Comparative Immunology* 24, 223–235. http://dx.doi.org/10.1016/S0145-305X(99)00074-9 10717289 10.1016/s0145-305x(99)00074-9

[CIT0044] Sheku K.M., 2013, ‘Appropriate time of vaccination against infectious bursal disease virus in layer chicks by Elisa in single dilution’, *Research Journal of Agricultural and Environmental Management* 2(4), 111–116.

[CIT0045] Shrestha P., Ahasan M.M., Islam K.M.D., Billah M.M., Islam M.E., Mehedi M. et al., 2003, ‘Sero-prevalence of infectious bursal disease virus (IBDV) specific antibody in chicken’, *Pakistan Journal of Biological Sciences* 6(14), 1234–1240. http://dx.doi.org/10.3923/pjbs.2003.1234.1240

[CIT0046] Suzuki K., Caballero J., Álvarez F., Faccioli M., Goreti M., Herrero M. et al., 2009, ‘Simulation models for estimating optimal vaccination timing for infectious bursal disease in broiler chickens in Paraguay’, *International Journal of Poultry Science* 8(6), 559–564. http://dx.doi.org/10.3923/ijps.2009.559.564

[CIT0047] Tsukamoto K., Tanimura N., Kakita S., Ota K., Mase M., Imai K. et al., 1995, ‘Efficacy of three live vaccines against highly virulent infectious bursal disease virus in chickens with or without maternal antibodies’, *Avian Diseases* 39, 218–229. http://dx.doi.org/10.2307/1591863 7677642

[CIT0048] Uddin M.M., Khan M.Z.I., Islam K.N., Kibria A.S.M.G., Adhikary G.N., Parvez M.N.H. et al., 2010, ‘Distribution of lymphocytes in the mucosa associated lymphoid tissues (MALT) of naturally occurring infectious bursal disease (IBD) in chicken’, *Pakistan Veterinary Journal* 30, 67–71.

[CIT0049] Van den Berg T.P. & Meulemans G., 1991, ‘Acute infectious bursal disease in poultry: Protection afforded by maternally derived antibodies and interference with live vaccination’, *Avian Pathology* 20, 409–421. http://dx.doi.org/10.1080/03079459108418779 18680037 10.1080/03079459108418779

[CIT0050] Wisniewska J. & Stosik M., 1999, ‘Serum antibody titer after the first immunization of broilers against IBDV’, *Medycyna Weterynaryjna* 55, 48–51.

[CIT0051] Wyeth P.J. & Cullen G.A., 1976, ‘Maternally derived antibodies effect on susceptibility of chicks to IBD’, *Avian Pathology* 5, 253–260. http://dx.doi.org/10.1080/03079457608418194 18777354 10.1080/03079457608418194

[CIT0052] Wyeth P.J. & Cullen G.A., 1979, ‘Use of an inactivated IBD oil emulsion vaccine in commercial broiler parent chickens’, *Veterinary Record* 104, 188–193. http://dx.doi.org/10.1136/vr.104.9.188 222040 10.1136/vr.104.9.188

[CIT0053] Zaheer A., Naeem K. & Malik S.A., 2003, ‘Comparative immune response pattern of commercial infectious bursal disease vaccine against field isolates in Pakistan’, *International Journal of Poultry Science* 2(6), 449–453. http://dx.doi.org/10.3923/ijps.2003.449.453

[CIT0054] Zaheer A. & Saeed A., 2003, ‘Role of maternal antibodies in protection against infectious bursal disease in commercial broilers’, *International Journal of Poultry Science* 2(4), 251–255. http://dx.doi.org/10.3923/ijps.2003.251.255

[CIT0055] Zhuo Z., Chen M., Zhou Z.Q. & Chen M.X., 1998, ‘Discussion on the causes for the outbreaks of IBD in immunized chicken flocks’, *Chinese Journal of Veterinary Medicine* 24, 14.

